# Sneddon’s syndrome: a comprehensive review of the literature

**DOI:** 10.1186/s13023-014-0215-4

**Published:** 2014-12-31

**Authors:** Shengjun Wu, Ziqi Xu, Hui Liang

**Affiliations:** Department of cardiothoracic surgery, The First Affiliated Hospital, School of Medicine, ZheJiang University, ZheJiang, China; Department of Neurology, The First Affiliated Hospital, School of Medicine, ZheJiang University, qingchun road 79, Hangzhou, ZheJiang China 310003

**Keywords:** Sneddon’s syndrome, Skin disease, Stroke, Systematic review

## Abstract

Sneddon’s syndrome (SS) is a rare non-inflammatory thrombotic vasculopathy characterized by the combination of cerebrovascular disease with livedo racemosa(LR). The Orpha number for SS is ORPHA820. It has been estimated that the incidence of SS is 4 per 1 million per annum in general population and generally occurs in women between the ages of 20 and 42 years. LR may precede the onset of stroke by years and the trunk and/or buttocks are involved in nearly all patients. The cerebrovascular manifestations are mostly secondary to ischemia (transient ischemic attacks and cerebral infarct). Other neurological symptoms range from headache, cerebral hemorrhage, seizures, cognitive and psychiatric disturbances. The involved internal organs include heart, kidney, and eyes. Histological findings of skin are characteristic and the involved vessels are small to medium-sized arteries at the border of dermis to subcutis with a distinct histopathological time course. The main diagnostic criteria are general LR with typical histopathological findings on skin biopsy and focal neurological deficits. The pathogenesis is related to hypercoagulable state and intrinsic small-vessel vasculopathy. The optimal management remains an unsolved problem and long-term anticoagulation have been recommended for cerebral ischemic events based on the presumed pathogenesis. There are controversial results in treatment of SS with immunomodulatory agents. The aim of this review is to comprehensively discuss this disease.

## Introduction

### Definition

Sneddon’s syndrome (SS) is a rare non-inflammatory thrombotic vasculopathy characterized by the combination of cerebrovascular disease with livedo racemosa (LR).

### History

The association between LR and cerebrovascular manifestation was first described by Kimming in 1959 [1]. In 1960, Champion and Rook described a case of cutaneous condition, diffuse arterial occlusive disease and cerebral ischemia [2]. 5 years later, Sneddon reported six patients with severe and generalized bluish discoloration of the skin (also termed as livedo reticularis), “multiple cerebrovascular incidents of limited and benign nature” and no autopsy reported [3]. Since then the term SS has been used to describe the association of LR and multiple cerebral infarcts.

### Classification

SS, which originally was a clinical diagnosis, is now regarded as a common clinical manifestation of different disease entities. Three forms of the syndrome were described by Schellong et al.: primary, where no causative factor could be identified, autoimmune with antiphospholipid antibodies (aPL) or coexisting systemic lupus erythematosus (SLE) or lupus-like disease and a thrombophilic form [4]. Francès et al. proposed that SS should be classified as idiopathic SS with neither aPL nor SLE, primary antiphospholipid syndrome (APS)-related SS, and SLE related SS with or without aPL [5]. Cases without any other pathological signs except LR should be historically labeled “idiopathic generalized LR” which may not exist as a separate entity but may represent a very early stage of SS.

### Epidemiology

While a number of SS cases have been reported since 1965, there is no definite evidence of ethnic differences in incidence. It has been estimated that the incidence of SS is 4 per 1 million per annum in general population [6]. In hospital-based series of stroke patients, the frequency of SS is between 0.25% and 0.50% [7,8]. A mortality rate of 9.5% is reported in a mean observation period of 6.2 years [6].

SS generally occurs in women between the ages of 20 and 42 years. For example, in the serial studies reported by Sneddon [3], 5 of 6 patients were women. Similarly, 34 of 46 patients were women reported by Francès and all cases were women in the report by Bolayir [5,9]. However, SS does occur occasionally in girls as young as 10 years old and in women as advanced as 64 years old [10].

### Genetic characteristics

The disorder mainly occurs sporadically, although a few familial cases have been reported [11]. Hademonos et al. [12] described in a review of the genetics of cerebrovascular disease and stroke that SS was inherited in an autosomal dominant pattern with unknown type of the gene and gene chromosome localization. In familial cases described by Rebollo et al. [13], either LR or full clinical SS was apparently transmitted in autosomal dominant pattern with complete or incomplete penetrance. The studies by Zhou et al. and Navon Elkan et al. have identified novel mutations in CECR1(cat eye syndrome chromosome region, candidate 1), encoding ADA2, as the cause of a syndrome including systemic vasculopathy and inflammation. Bras etal. extend the phenotypic spectrum of ADA2 deficiency to include a familial form of SS [14,15].

### Clinical description

#### Dermatologic manifestations

**Figure 1:**
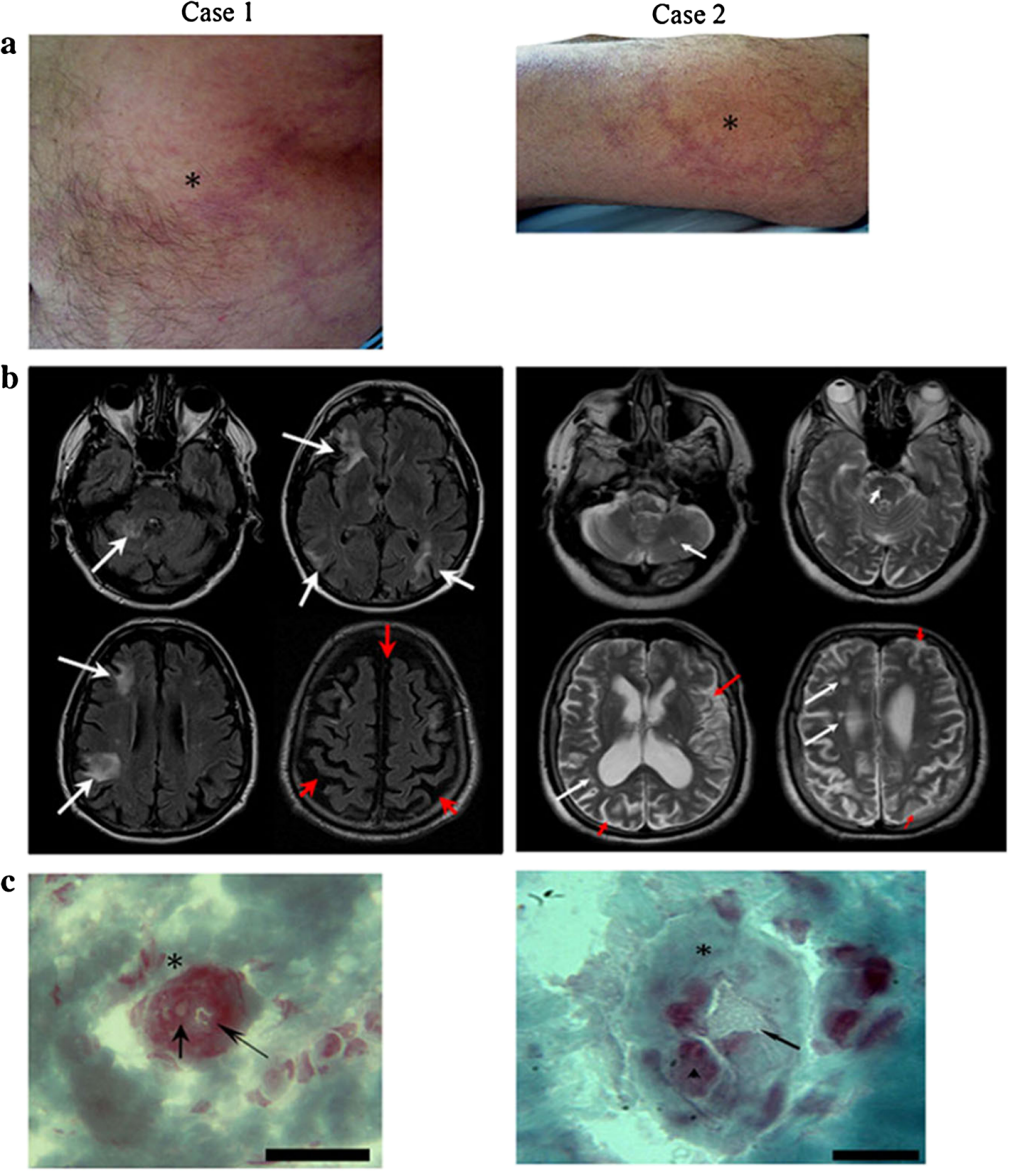
**(two cases of SS ) a, diffused livedo racemosa (asterisks); b, FLAIR/T2 MRI brain scan showing multiple ischemic lesions (white arrows) and cortical atrophy (red arrows); c, skin biopsy showed intra-capillary (thin arrow) and parietal (thick arrow) widespread thrombosis; scale bar 100μm (Case1) and 50 μm (Case2).** (courtesy of Dr. Alessandro Tessitore).

One of the diagnostic hallmarks of SS is LR (Figure 1a). LR is defined as a dusky erythematousto-violaceous, irregular, net-like pattern in the skin. LR may precede the onset of stroke by years and is located on limbs (100% each), trunk (84–89%), buttocks (68–74%), face (15–16%), or the hands or feet (53–59%) [5]. The trunk and/or buttocks are involved in nearly all patients. LR is noticed before cerebrovascular events in more than a half of patients. In some patients, the livedo is first detected at the time of stroke occurrence [16,17]. In rare cases, LR appears after neurological symptoms [18]. In addition to LR, some patients have signs of Raynaud’s phenomenon involving hands and feet and some have widespread cutaneous, mottled-purple discoloration on the body which had been diagnosed as systemic angiomatosis [19,20].

LR is similar to the familiar sign of livedo reticularis from which it differs by its shape (irregular, broken circular segments, resulting in a seemingly larger pattern), and persistence on warming [21,22]. In the American literature, livedo reticularis is used interchangeably with LR despite their different pathophysiological basis. Livedo reticularis is caused by temporary vasoconstriction, whereas livedo racemosa results from persistent impairment of peripheral blood flow caused by occlusion of small or medium-sized arteries [23,24].

#### Neurological manifestations

Stroke is another diagnostic hallmark of SS. This cerebrovascular disease mostly occurs due to ischemia (transient ischemic attacks and cerebral infarct) in the perfusion zones of middle cerebral artery or posterior cerebral artery [25,26]. Hemiparesis, sensory disturbances, aphasia and visual field defects are the most common clinical signs. However, most patients are minor stroke. Headache represents the most frequent unspecific symptom [27,28]. The 50% of these patients have migrainous headache [29]. The frequency of headache is not significantly higher in persons with positive aPL when compared to an aPL-negative cohort [29].

Cognitive impairment and psychiatric disturbances can occur in approximately 77% of SS patients and SS can also be a cause of dementia in the young [25,30]. These patients develop cognitive dysfunction over years and then dementia due to the cumulative effects of multiple cerebral infarcts. Because of these features, SS may lead to psychiatric disturbances, including depression. Concentration, attention, memory, visual perception and visuospatial construction are the most commonly described cognitive dysfunctions [31]. However, patients with SS can develop topographic disorientation or dementia without antecedent clinical stroke [18]. Focal or secondary generalized seizures are commonly seen in SS, especially in patients with positive aPL [5,32].

Intracerebral (ICH), subarachnoid or intraventricular hemorrhage are unusual in SS. Movement disorders including chorea and tremor were occasionally been described [33,34].

#### Other manifestations

Hypertension occurs in a significant proportion (15%–65%) of SS patients [25,26]. Heart valvulopathy was found in 41% of SS patients in one study and 61% in another study [35,26]. Mitral valve thickening has been revealed more frequently in patients with aPL than in those without. Cases of SS with ischemic heart disease are described including patient associated with successful percutaneous coronary intervention [36]. The renal function is slowly deteriorated in SS. Zelger et al. reported a long-term follow-up of 21 patients and observed that 65% of patients showed decreased levels of creatinine clearance [6].

Ophthalmologic complications include central retinal artery occlusion, central retinal vein occlusion, retinal neovascularization, homonymous visual field defects, and internuclear ophthalmoloplegia [37-42]. Rehany et al. reported a young SS patient with internuclear ophtalmoplegia followed by ophthalmic artery occlusion [41]. Unilateral third cranial nerve palsy has been seen in a patient with SS and this is likely associated with microvascular complication [43].

#### Etiology

The precise etiology is unknown and around 50% of cases are idiopathic. Female reproductive hormones, oral contraceptives and hypertension are correlated with disease progression. The frequent presence of aPL suggest that SS is part of the clinical spectrum of the primary aPL syndrome. The proportion of aPL antibodies is widely different in various cohorts of SS [6,35,44]. The majority authors suggest that 40–50% of SS patients are aPL positive [5,45]. However, some patients are aPL-negative which indicate that this syndrome may be a distinct entity or perhaps a group of different disorders.

### Pathology

#### Dermatopathology

Skin biopsy is critically important for the early diagnosis of SS. Skin biopsy may reveal thrombosis of subcutaneous arterioles and compensatory capillary dilation with blood stagnation causing LR (Figure 1c). Several lines of evidence showed endarteritis obliterans from intimal endothelial proliferation and proliferation of medial smooth muscle cells [24,46,47], although skin biopsies were negative or non-specific in some cases [1,48]. Pathogenesis of SS has been proposed a stepwise pattern of disease progression by Sepp et al. and Zelger et al. [49,50], beginning as an inflammatory vascular process in small to medium sizes of arteries. After the initial phase (stage I) of endotheliitis, characterised by a plug, the formation of the plug of lymphohistiocytic cells and fibrin would occur in the early phase (II stage), followed by the intermediate phase (stage III) of subendothelial migration of smooth muscle cells, and completed by the late phase (stage IV) of vessel shrinkage and fibrosis. Skin biopsy may reveal different pathological phases in one patient. However, the severity of the clinical findings is not normally correlated with the histological findings. Deep skin biopsies increase sensitivity from 27% with one biopsy, to 53% with two biopsies, and to 80% with three biopsies taken from white areas in the most of cases [51].

#### Brain pathology

Brain biopsy is rarely done in SS. The pathologic findings showed multiple vascular thrombosis without vasculitis in the first brain biopsy case [52]. There were cortical infarcts and leukoencephalopathy associated with intimal fibrosis of the basal vessels in a biopsy report [53]. The other autopsy report described a diffuse vasculopathy affecting small to medium sized arteries (including cerebral vessels) characterized by proliferation of the intima and media, with some vessels showing fibrotic occlusion and recanalization. Inflammation did not affect blood vessels [54]. The principal pathologic findings in the case reported by Hilton DA et al. were multiple small cortical infarcts associated with occlusion of medium sized arteries and prominent focal smooth muscle hyperplasia of smaller arterial vessels [55]. Although a granulomatous reaction in the leptomeninges has been reported [56], this reaction is not observed by others [54], suggesting that this abnormality does not always occur in SS.

#### Pathophysiology

Pathophysiology of SS remains incompletely understood to date. The existence of aPL antibodies suggests that symptoms are secondary to thrombotic process [57]. Vascular thrombosis and recanalization within skin and brain tissue support the pathophysiology of coagulopathy.

Although a basic thrombotic process may appear in SS, it is still not clear how it occurs in aPL-negative cases.Various abnormalities have been reported in isolated cases: activated protein C resistance [58], platelet aggregability [49], increased thromboglobulin levels [59], modifications of the ratio tissue plasminogen activator/inhibitor [60], familial deficiency in antithrombin III [46], and protein S deficiency [61]. None of these abnormalities have been confirmed in large series of studies. Protein Z, a downregulator of coagulation and linked to an increased risk of arterial thrombosis, is significantly lowered in patients with aPL-negative SS [62]. Heart valve abnormalities, either clinically audible or detected on echocardiogram, are frequently observed, and their total prevalence is similar in both groups. This raises the possibility that the embolic mechanism plays an important role in the occurrence of some neurologic and even skin manifestations of SS [63].

In some patients, no aPL or primary coagulation deficits are detected. Thus, the primary vasculopathy is considered as the pathophysiological change in SS. It is speculated that a nonvasculitic small and medium sized vessel arteriopathy causes both skin symptoms and cerebrovascular events [48]. However, the type and origin of the arteriopathy is largely unknown [64]. In some cases, the gene responsible for the arteriopathy may play a role [41,65]. Besides, endothelial dysfunction may be secondary to acquired autoimmune or mediated by some unknown factors [66,67].

#### Diagnosis

Any patient suspected of SS should undergo blood tests, thorough cardiovascular evaluation, cerebral MRI, cerebral angiography, and skin biopsy. Blood tests should screen for lupus anticoagulant, immunoglobulin IgG and possibly IgM anti-cardiolipinantibodies, anti-nuclear and anti-double-stranded DNA autoantibodies, thrombocytopenia, leukopenia, VDRL, cryoglobulins, circulating immune complexes, antithrombin-III, protein C, or protein S [3,13,68,46]. The cerebrospinal fluid (CSF) is usually normal.

Lesions can be more clearly detected by MRI than by CT scan. These lesions are often small and multifocal, located predominantly in the periventricular deep white matter or pons (Figure 1b). Cerebral hemorrhages are rarely found in SS. Prominent microbleeds are found in familial SS, which might be a marker of impending haemorrhagic stroke [65,69]. Cerebral angiography is abnormal up to 75% of patients with SS. The most common abnormality is an obliterating noninflammatory arteriopathy with stenosis and/or occlusion of intracranial vessels [70-73]. Other reported findings include: arteriovenous malformations involving meningeal branches, transdural anastomoses, granulomatous leptomeningeal infiltration, and a large network of fine collateral vessels [70-76]. Occurrence of an aneurysmatic dilatation of the ascending aorta is rare in SS [77].

The main criteria are general LR with typical histopathological findings on skin biopsy and focal neurological deficits. Supportive criteria are a history of transient ischemic attacks or stroke and evidence of small bright foci on T2-weighted MRI [68].

#### Differential diagnosis

Some diseases that can occur with generalized LR (such as SLE, polyarteritis nodosa, cryoglobulinemia, livedoid vasculitis, cold agglutinin disease, arteriosclerosis, cholesterin embolization and others [6,22,59]), must be excluded by appropriate clinical and laboratory tests. The differential diagnosis of neurological symptoms include multiple sclerosis, multiple embolization from cardiac source, infectious conditions (such as syphilis and lyme disease), and others [6,43]. Central nervous system (CNS) vasculitis refers to a broad array of diseases that result in inflammation and destruction of the blood vessels of brain, spinal cord and the meninges. CNS vasculitis is classified into systemic vasculitis and isolated CNS angiitis. CNS vasculitis may present symptoms such as headache and stroke which mimic SS patients. However, in systemic vasculitis, CNS involvement always coexists with other clearly apparent systemic manifestations. Isolated CNS angiitis is twice as frequent in males as in females and onset most often occurs after 40 years of age. The sedimentation rate is moderately increased in about 30% patients and CSF inflammation is observed in about 90% of patients. Histological findings are characterized by infiltrations of the vascular walls with mononuclear cells including lymphocytes, macrophages, and histiocytes [78].

#### Treatment

The optimal management of patients with SS remains an unsolved issue and controlled trials have not yet been performed. Although nifedipine may reduce skin symptoms, it does not prevent cerebrovascular complications [6]. Based on the presumed pathogenesis of SS, some researchers have recommended long-term anticoagulation for cerebral ischemic events [79,80]. Khamashta et al. reported in a retrospective study of 183 patients with APS syndrome that the number of events per year of follow up was 0.18 with aspirin, 0.23 with low dose warfarin (INR < 3), and 0.015 with high-dose warfarin (INR ≥ 3). In a retrospective study, Francès et al. found that antiplatelet therapy was less effective in aPL-positive than in aPL-negative patients. Among the former, the number of recurrent cerebral events per year of follow-up was 0.5 with antiplatelet therapy and 0.06 with anticoagulation. The three aPL-positive patients who experienced a new cerebral ischemic event under anticoagulation had an INR less than 3 at the time of recurrence [5]. The benefits of warfarin in the APS are greater than the risks of bleeding [79].

In SS patients with acute ischemic stroke, thrombolytic therapy might be safe and effective [81]. Angiotensin converting enzyme inhibitors (ACEI) and prostaglandin E1 (PGE1) have also been used in the treatment of SS [82]. The prevention of smoking and prevention of usage of estrogen oral contraceptive may prevent or decrease the severity of neurological symptoms [6]. Moreover, cardiovascular risk factors should also be treated.

Corticosteroids, cyclophosphamide, and azathioprine have been reported ineffective in treatment of SS [83]. However, one study demonstrated improvements in neurological and cognitive symptoms in a patient after 8 months of monthly intravenous cyclophosphamide therapy [73]. Future research should focus on identifying potential subgroups of patients who may be good responders.

## Conclusion

SS is a clinically syndrome that probably stems from a number of acquired or congenital hemostatic abnormalities which preferentially involves the cerebral and cutaneous vascular beds. To a certain extent, the small number of cases may reflect the unfamiliarity with the syndrome rather than a true reflection of its incidence. Further research exploring the pathogenesis of SS is needed to get more etiological subgroups. Future therapy should identify different treatment modalities for different etiological subgroups.

## Abbreviations

SS: Sneddon’s syndrome
LR: Livedo racemosa
aPL: Antiphospholipid antibodies
SLE: Systemic lupus erythematosus
APS: Antiphospholipid syndrome
ACEI: Angiotensin converting enzyme inhibitors
PGE1: Prostaglandin E1
